# Translating transmissibility measures into recommendations for coronavirus prevention

**DOI:** 10.11606/s1518-8787.2020054002471

**Published:** 2020-04-03

**Authors:** Fredi Alexander Diaz-Quijano, Alfonso Javier Rodriguez-Morales, Eliseu Alves Waldman

**Affiliations:** I University of São Paulo School of Public Health Department of Epidemiology São PauloSP Brazil University of São Paulo. School of Public Health. Department of Epidemiology. São Paulo, SP, Brazil; II University of São Paulo School of Public Health Laboratório de Inferência Causal em Epidemiologia da Universidade de São Paulo São PauloSP Brazil University of São Paulo. School of Public Health. Laboratório de Inferência Causal em Epidemiologia da Universidade de São Paulo (LINCE-USP). São Paulo, SP, Brazil; III Universidad Tecnologica de Pereira Faculty of Health Sciences PereiraRisaralda Colombia Universidad Tecnologica de Pereira. Faculty of Health Sciences. Public Health and Infection Research Group. Pereira, Risaralda, Colombia; IV Fundación Universitaria Autónoma de las América Faculty of Medicine PereiraRisaralda Colombia Fundación Universitaria Autónoma de las Américas. Faculty of Medicine. Grupo de Investigación Biomedicina. Pereira, Risaralda, Colombia

**Keywords:** Coronavirus Infections, epidemiology, Disease Transmission, Infectious, prevention & control, Epidemiological Monitoring, Health Communication, COVID-19, Basic reproductive number

## Abstract

The rapid increase in clinical cases of the new coronavirus disease, COVID-19, suggests high transmissibility. However, the estimates of the basic reproductive number reported in the literature vary widely. Considering this, we drew the function of contact-rate reduction required to control the transmission from both detectable and undetectable sources. Based on this, we offer a set of recommendations for symptomatic and asymptomatic populations during the current pandemic. Understanding the dynamics of transmission is essential to support government decisions and improve the community’s adherence to preventive measures.

## INTRODUCTION

The rapid increase in clinical cases of the new coronavirus disease, COVID-19, suggests that its transmissibility is high. Several studies indicated that the most probable basic reproductive number (*R*_0_) is between two and four for the coronavirus (meaning that each infectious individual may directly generate two to four others)^1^. However, some estimates suggested *R*_0_ higher than four with confidence intervals that reach values close to eight^2,7^. A reproductive number higher than one implies a progressive increase in cases, which translates into epidemics. Therefore, the reduction in this value must guide preventive strategies towards transmission control. In this study, we applied some simple calculations to translate the transmissibility measures into goals for contact rate reduction to support preventive recommendations during the coronavirus pandemic.

## METHODS AND RESULTS

The *R*_0_ results from the product of three factors, such as *R*_0_ = *dpc*; where *d* is the duration of infectiousness (the period when a case can be a source of transmission), *p* is the probability of transmission by contact, and *c* is the contact rate^8^. The duration of infectiousness and the transmission probability by contact are practically inherent to the pathology and, therefore, difficult to modify. However, the contact rate is more feasible to control^9^.

A theoretically effective strategy would be to eliminate any kind of contact between people, which is impractical. Therefore, a reasonable goal is to reduce contacts to the point that the reproductive number is lower than one. If we consider a *R*_0_ of eight, it would be necessary to minimize the contact rate for each infectious case by about $90\%\;\left(>\;87.5\%,\;i.e.,\;>\;1\;-\;\frac{1}{R_{0}}\right)$. As it is difficult to test all the suspected patients, a sensible recommendation during the epidemic is to apply this goal to all people with respiratory symptoms, which means domestic confinement as long as they do not need specialized care.

Asymptomatic people must also be considered because they represent the majority of infections. Recent studies suggested that about 86% of infections can be undocumented. Although the transmission probability from them appears to be 45% lower than from documented cases, undocumented infections are the source of 79% of clinical cases^10^. Given the limitations of both logistics and resources to carry out mass testing, in epidemic situations, everyone must be considered a potential carrier of the infection. Thus, a reasonable goal for asymptomatic people is to reduce the contact rate by more than $78\%\;\left(>\;77.3\%,\;i.e.,\;1\;-\;\frac{1}{0.55R_{0}}\right)$.

These estimates were based on extrapolations from different studies^2,7,10^, for which we assumed the worst-case scenarios. Considering this, we represented the relationship between the baseline *R*_0_ of identifiable cases and the minimum necessary reduction in their contact rate to decrease that reproductive number to less than one (solid line in the figure). Additionally, we represented the minimum contact-rate reduction that should be concomitantly adopted by the undetectable infection cases to achieve the same goal (dotted line). According to these functions, even in a more optimistic scenario, for example, with a *R*_0_ of 4, contact rate should be reduced by at least 75% and 55% for symptomatic and asymptomatic people, respectively (Figure).

**Figure:**
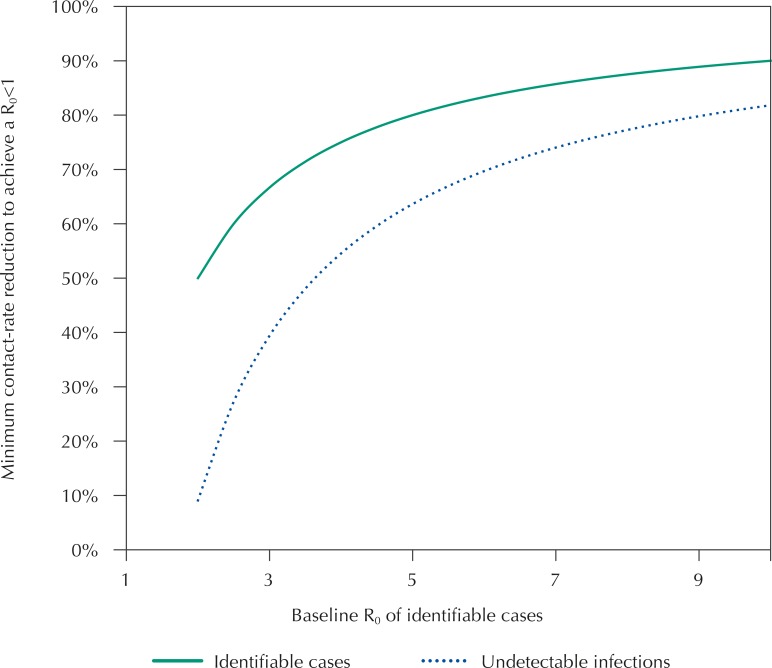
The minimum contact-rate reduction required according to the basic reproductive number (R0).

## DISCUSSION

Although it is challenging to quantify how many contacts people usually have, the estimates presented help to understand the need for physical isolation, even among asymptomatic people. The minimum contact-rate reduction refers to that necessary only to decelerate the increasing transmission trend. Consequently, to achieve effective control, reduction must far exceed the minimum recommended and involve the entire community at risk.

The virus is transmitted by aerosols that could remain suspended in the air for many minutes after coughing or sneezing^11^. Moreover, it can remain viable for a few days on multiple surfaces. Therefore, proper cleaning of shared spaces and personal hygiene are critical. During the epidemic, even asymptomatic people should avoid crowding and reduce all activities that imply proximity to others, including public transportation, as well as social, cultural, and academic events.

Whenever possible, work and study activities should be carried out virtually, and commercial activities and travel should be reduced to what is strictly essential, such as the purchase of food or medicines. Cases with mild respiratory symptoms should be treated at home with hydration and paracetamol when needed. Visits to health centers should only be made when justified by the presence of risk factors (underlying severe disease) or warning signs (such as breathing difficulties or cognitive impairment).

Older patients are especially susceptible to complications^12^. However, to reduce transmission, people of all ages must be committed to prevention. Thus, in the absence of vaccines or other preventive strategies, reducing the contact rate will be the only strategy to slow the progression of this pandemic.

If these measures are adequately applied, the number of new cases is expected to decrease. It is difficult to predict for how long these preventive measures must be maintained. Nevertheless, the earlier and more rigorously they are incorporated, the faster the epidemiological conditions will be reversed and our regular routines resumed.
